# BubR1 and brain aging

**DOI:** 10.18632/aging.101300

**Published:** 2017-10-02

**Authors:** Syed Mohammed Qasim Hussaini, Mi-Hyeon Jang

**Affiliations:** Department of Neurologic Surgery, Mayo Clinic College of Medicine, Rochester, MN 55905, USA

**Keywords:** BubR1, hippocampus, aging

The hippocampus is one neurogenic region in the adult mammalian brain that continues to produce neurons well into adulthood. This process of neurogenesis occurs in the subgranular zone (SGZ) of the hippo-campal dentate gyrus that harbors neural stem cells (NSCs). These actively participate in a sequential process where they proliferate, migrate and mature into neurons that are functionally integrated into the hippocampal circuitry [1]. This is a highly plastic process that affords the hippocampus roles in memory formation, learning, and mood regulation. However, it is also an age-dependent one where the number of NSCs decline with age. Age-related cognitive disability is one example of the functional implications of deficits in this process. A molecular understanding of this course has so far eluded the field. Recent evidence has demonstrated that BubR1, a mitotic checkpoint kinase, decreases with natural aging and induces progeroid features [2] and aging-related CNS abnormalities [3]. In our recent study in Aging Cell, we sought to address if BubR1 played a role in age-related hippocampal changes [4].

In this study, we show BubR1 is expressed in the radial-glia like NSCs (RGC), and its expression is reduced in an age-dependent manner. We used progeroid BubR1^H/H^ mice with reduced hippocampal BubR1 levels to show significantly reduced proliferation. Progenitor cell types vulnerable to BubR1 insufficiency included significant reductions in activated RGCs, intermediate progenitor cells, and neuroblasts. Such changes in cellular proliferation were exacerbated in *BubR1* ^H/H^ mice in an age‐dependent manner. Next, we sought to address if BubR1 played a role in maturation of the surviving neurons. An *in vitro* analysis using post-mitotic neurons derived from adult NSCs showed BubR1 localization in the dendrites and the cytoplasm. Using an EdU pulse chase, BubR1^H/H^ mice showed a significant increase in the portion of immature neurons with a concurrent decrease in mature neurons, indicat-ing delayed neuronal maturation in BubR1^H/H^ mice. Using a shRNA-BubR1 retroviral approach, we could label and selectively knock down BubR1 within these new neurons. At 14 days post-injection, shRNA-BubR1 compared with shRNA-control mice showed sig-nificantly decreased primary dendrite length, total dendrite length, and branch number. Importantly, these morphological alterations were significantly rescued in BubR1-overexpression mice, suggesting a critical post-mitotic role of BubR1 in neuronal morphogenesis of newborn neurons.

This study expands on the varied and emerging functions of BubR1 and implicates it as a key regulator in the age-dependent changes in adult hippocampal neurogenesis. In addition, while BubR1 is primarily known as a key component for mitosis [5], our study is the first to delineate the critical post-mitotic role for BubR1 in neuronal maturation and morphogenesis of newborn neurons. However, this study does not yet provide the mechanistic link or elucidation of the molecular machinery that occurs between BubR1 decrease and significant reductions in proliferation and maturation of newborn hippocampal neurons. Recent studies from our lab have identified involvement of Wnt signaling as a novel molecular regulator to this process (*unpublished)*. Furthermore, it remains to be understood if sustained BubR1 levels during aging process may have a protective role in the aged brain, and thus represent a novel therapeutic target for age-related cognitive declines. This is a future direction that can shed further light on BubR1 and aging.

The elderly population is rapidly growing worldwide. This increase in lifespan has been accompanied by an increased frequency of cognitive impairments, Alzheimer's disease, and specific age-related comor-bidities such as vascular disease that significantly alter one's quality of life. Understanding the neurobiological mechanisms of aging and cognitive impairments can bolster our ability to design new treatments to help an increasingly elderly population. To this end, the hippocampus has been of unique interest to the scientific community with its roles in neurogenesis and memory formation owing to its inherent neuroplasticity. Behavioral experiments in animal models have provided an additional way to investigate previously difficult to evaluate functions such as episodic memory [6]. Evidence from animal models has demonstrated how structural and cellular-level changes in the hippocampus can result in cognitive impairments. While such changes have been well-characterized, it has been more difficult to evaluate the molecular machinery driving this process. In our recent study, we have shown BubR1 playing a crucial role in regulating age-related dysfunction in axon myelination and motor function [3]. In the current study, we expand its role to hippocampal neurogenesis. Our study fits into a larger effort to understand the drivers of hippocampal neurogenesis and how these impact aging and cognitive deficits. Considering the emerging role of BubR1 in the patho-biology of aging, and studies demonstrating how high levels of BubR1 extend lifespan and delay age-related deterioration, we believe the findings of our present study provide novel insight into how this process may be regulated. It has broad implications in, and opens avenues for further exploration of aging-related neuro-degenerative diseases and therapeutic approaches to treat such diseases.
